# Contribution of Dysregulated B-Cells and IgE Antibody Responses to Multiple Sclerosis

**DOI:** 10.3389/fimmu.2022.900117

**Published:** 2022-06-16

**Authors:** Malik R. Seals, Monica M. Moran, Jonathan D. Leavenworth, Jianmei W. Leavenworth

**Affiliations:** ^1^ Department of Neurosurgery, University of Alabama at Birmingham, Birmingham, AL, United States; ^2^ Multidisciplinary Biomedical Sciences, University of Alabama at Birmingham, Birmingham, AL, United States; ^3^ Graduate Biomedical Sciences Program, University of Alabama at Birmingham, Birmingham, AL, United States; ^4^ Department of Dermatology, University of Alabama at Birmingham, Birmingham, AL, United States; ^5^ Department of Microbiology, University of Alabama at Birmingham, Birmingham, AL, United States; ^6^ The O’Neal Comprehensive Cancer Center, University of Alabama at Birmingham, Birmingham, AL, United States

**Keywords:** B-cells, humoral antibody response, IgE, macrophage, microglia, neuroinflammation, multiple sclerosis, experimental autoimmune encephalomyelitis

## Abstract

Multiple sclerosis (MS), a debilitating autoimmune inflammatory disease that affects the brain and spinal cord, causes demyelination of neurons, axonal damage, and neurodegeneration. MS and the murine experimental autoimmune encephalomyelitis (EAE) model have been viewed mainly as T-cell-mediated diseases. Emerging data have suggested the contribution of B-cells and autoantibodies to the disease progression. However, the underlying mechanisms by which dysregulated B-cells and antibody response promote MS and EAE remain largely unclear. Here, we provide an updated review of this specific subject by including B-cell biology and the role of B-cells in triggering autoimmune neuroinflammation with a focus on the regulation of antibody-producing B-cells. We will then discuss the role of a specific type of antibody, IgE, as it relates to the potential regulation of microglia and macrophage activation, autoimmunity and MS/EAE development. This knowledge can be utilized to develop new and effective therapeutic approaches to MS, which fits the scope of the Research Topic “Immune Mechanism in White Matter Lesions: Clinical and Pathophysiological Implications”.

## Introduction

Multiple sclerosis (MS) is the most prevalent chronic inflammatory demyelinating disease of the central nervous system (CNS), affecting approximately 2.8 million people worldwide (1, 2). MS varies between patients at presentation, displaying as one of three clinical forms, primary-progressive MS (PPMS), secondary-progressive MS (SPMS) and relapsing-remitting MS (RRMS). Approximately 85% of MS patients are diagnosed with RRMS, which is typically characterized by acute, episodic periods of aggressive symptomology (relapses), followed by periods of remission (3). Symptoms of MS include, but are not limited to, sensory loss, fatigue, visual impairments and ataxia in consequence of inflammation, neuron demyelination and the accumulation of white-matter lesions in the CNS. The progression of these disease manifestations consequently results in gradual loss of mobility and cognition potentially leading to neurologic disability (2). Currently, no medications can prevent or reverse neurological deterioration (2).

MS is generally classified as an immune-mediated inflammatory disease, involving a complex combination of neurodegenerative processes amplified by immunological responses. During a relapse, immune cells are activated in the periphery and traffic into the CNS, where they are re-activated, trigger inflammation and recruitment of other peripheral immune cells, consequently resulting in neuron demyelination and tissue damage (3). The C57BL/6 (B6) murine experimental autoimmune encephalomyelitis (EAE) is the most widely used experimental model of MS due to the availability of transgenic and gene knock-out models on the B6 background that are used to investigate the disease pathogenesis (referring to reference 5 for details of other EAE models). The model is established by sensitizing mice to myelin oligodendrocyte glycoprotein MOG_35-55_ peptide emulsified in complete Freund’s adjuvant along with the co-adjuvant pertussis toxin (4). It can be actively induced through direct immunization, or passively through adoptive transfer of myelin-specific T-cells or CD4^+^ T helper (T_H_) cells from a sensitized donor to a naïve recipient (5). EAE is characterized by the infiltration of autoreactive immune cells into the CNS with subsequent inflammation, demyelination, axonal damage and loss. Due to the potential immune mechanisms involved in both MS and murine MOG_35-55_-induced EAE, this model remains important in the MS research, despite that the lesions in this model, which are different from the MS pathology, appear to display extensive axonal injury and loss with little primary demyelination and are largely confined to the spinal cord (5).

Extensive studies on EAE and MS have focused on myelin-specific autoreactive T-cells. The involvement of B-cells in MS has also been appreciated, as evidenced by the presence of oligoclonal bands (OCB) in the cerebral spinal fluid (CSF) of MS patients (2, 6–8), and the effectiveness of recent B-cell depleting therapies in MS patients that highlights antibody-independent B-cell function in MS pathogenesis (9–12). The primary focus of this review will discuss our expanded understanding of B-cell involvement in autoimmune neuroinflammation with a focus on antibody-secreting B-cells. We will then discuss the role of a specific type of antibody, IgE, as it relates to autoimmunity and MS/EAE development.

## Biology of B-Cells

The role of B-cells in the maintenance of proper immune function and regulation displays profound importance in human health and disease. B-cells as a highly heterogenous population with a great degree of functional plasticity are capable of modulating both innate and adaptive immune responses. Consequently, their development and effector functions are highly regulated, as an inappropriate development can lead to the generation of autoreactive B-cells (13).

### B-Cell Developmental Subsets

B-cells develop from hematopoietic stem cells in the bone marrow (BM), supported by growth factors and cytokines produced by resident stromal cells (14). The early B-cell development in the BM involves different stages and rearrangements of immunoglobulin (Ig) heavy and light gene segments by recombination activating gene-dependent processes, resulting in an enormous diversity of the antibody repertoire (15). In mice, these early developing B-cells finally differentiate into transitional B-cells which then migrate from the BM mainly to the spleen, while in humans transitional B-cells are present in the circulation and secondary lymphoid organs (SLOs), including the spleen (16). The late transitional B-cells give rise to mature B-cells, including follicular and marginal zone (MZ) B-cells. Upon activation, both follicular and MZ B-cells can develop into antibody-secreting cells (ASCs), including cycling plasmablasts and non-dividing plasma cells, in a T-cell-independent manner (17). When follicular B-cells encounter antigen recognition and T-cell help, they can also be fully activated, migrate into the follicle and induce the germinal center (GC) formation in SLOs (17). The cognate T-B interactions, namely T follicular helper (T_FH_)-GC B-cell interactions, in the GC facilitate GC B-cells to undergo Ig class switch recombination and somatic hypermutation followed by developing into memory B-cells or ASCs, including long-lived plasma cells producing class-switched antibodies (14, 16). Memory B-cells are primarily responsible for mounting the secondary immune response, triggered following a subsequent exposure to the same antigen they recognize for the first time, allowing them to proliferate and differentiate into plasma cells (18).

### Normal B-Cell Functions

A key characteristic of the mammalian adaptive immune response is the ability to rapidly produce and secrete high-affinity antibodies as they detect and help clear foreign pathogens or generate protective immunity to vaccination. B-cells are indispensable for the generation of plasmablasts and plasma cells, which are the only cell types capable of antibody production. There are five distinct antibody isotypes: IgA, IgD, IgE, IgG, and IgM, each possessing a unique effector function and distinct heavy chain. Each antibody contains a unique amino acid sequence and antigen-binding site that resides in the antigen-binding fragment (Fab) or the variable fragment (Fv) of Fab, conferring antigen specificity (19). Antibodies serve three primary functions: neutralization, activation of the complement cascade, and initiation of pathogen opsonization. Neutralization occurs when secreted antibodies bind to infectious pathogens and toxins, rendering them ineffective. Alternatively, antibodies can activate the complement cascade, a complex innate immune surveillance system that plays a primary role in protection against bacterial pathogens. Lastly, antibodies can initiate opsonization upon binding the foreign target, flagging it for destruction by phagocytic cells (20).

Although B-cells are most notable for their ability to generate and secrete antibodies, they also provide several other key effector functions, including antigen presentation, directing immune responses through cytokine secretion, and immunoregulatory roles. B-cells, recognized as professional antigen-presenting cells (APCs), constitutively express major histocompatibility complex class II (MHCII) molecules produced during B-cell development in the BM. MHCII expression in mature B-cells permits the presentation of antigens to naïve CD4^+^ T-cells, which triggers T-cell activation and proliferation when coupled with co-stimulatory signals (21). B-cells also possess the ability to modulate immune responses. Effector B-cells, Be1 or Be2 subsets, arising from naïve B-cells upon activation can influence CD4^+^ T-cell differentiation into effector and memory subtypes through distinct cytokine production. T_H_1 differentiation is promoted by Be1 cells that secrete tumor necrosis factor (TNF-α), interferon-γ (IFNγ), interleukin-12 (IL-12), and IL-6, while T_H_2 differentiation is driven by Be2 cells producing IL-2, IL-13 and IL-4 (22–24). IL-6-secreting B-cells are also implicated in the development of autoimmunity and promote the development of proinflammatory T_H_17 cells, a cell subset known to play a crucial role in MS pathogenesis (25). Finally, B regulatory cells (Bregs) can negatively regulate immune responses through the production of anti-inflammatory cytokines, IL-10, IL-35 and transforming growth factor β (TGF-β) (22, 26–28). Bregs also play an important role in maintaining invariant natural killer T-cells, which promote tolerance against autoantigens involved in autoimmune diseases (29). A continuous effort is being made to understand Breg properties and functions in healthy adults as a means of developing effective immunotherapies.

### Regulation of GC B-Cell Responses

Upon activation, B-cells undergo differentiation into ASCs that are classified as either plasmablasts or plasma cells (30). ASCs can be generated *via* an extrafollicular differentiation pathway and produce relatively low-affinity antibodies. Here we focus on ASCs derived from the GC, where high-affinity antibodies and memory B-cells are generated (17).

Within the GC reaction, somatic hypermutation of the variable regions of Ig genes allows B-cells to produce high-affinity antibodies. However, random point mutations can also lead to nonfunctional or autoreactive antibody production, thus this process is tightly regulated by positive and negative selection mechanisms controlling for antibody affinity, while avoiding autoreactivity (31, 32). Two critical players in these regulatory processes include T_FH_ and T follicular regulatory (T_FR_) cells. T_FH_ cells are a specialized subset of CD4^+^ T_H_ cells, characterized by the expression of programmed cell death protein 1 (PD-1), chemokine receptor CXCR5 and B cell lymphoma 6 (Bcl6) transcription factor (33–36). T_FH_ cells are localized in B-cell follicles, where they participate in the GC formation, promote the proliferation and differentiation of GC B-cells, and support the production of high-affinity antibodies *via* the cognate interactions with GC B-cells. T_FH_-derived IL-21 can promote GC responses by directly acting on B-cells while simultaneously promoting Bcl6 expression to further enhance T_FH_ cell development (35–37). During affinity maturation, B-cells undergo division and Ig gene mutation in the GC dark zone followed by migrating to the light zone, where B-cells with higher affinity receptors present antigens to T_FH_ cells for selection of high-affinity cells that cycle back to the dark zone for additional rounds of division and mutation (38, 39). This T_FH_-mediated selection process is accompanied by substantial cell death and apoptosis, which potentially provides self-antigens and induces autoreactive antibody production (40, 41), testifying a need for a tight regulation of this process. Although T_FR_ cells share surface features with T_FH_ cells and are also Bcl6^+^, they are the immunosuppressive counterpart of T_FH_ cells, as a subset of Foxp3^+^ regulatory T-cells (Tregs) with their primary role to control GC responses and maintain humoral self-tolerance (42–45). A precise control of GC responses requires the maintenance of the balance between T_FH_ and T_FR_ cell responses.

**Figure 1:**
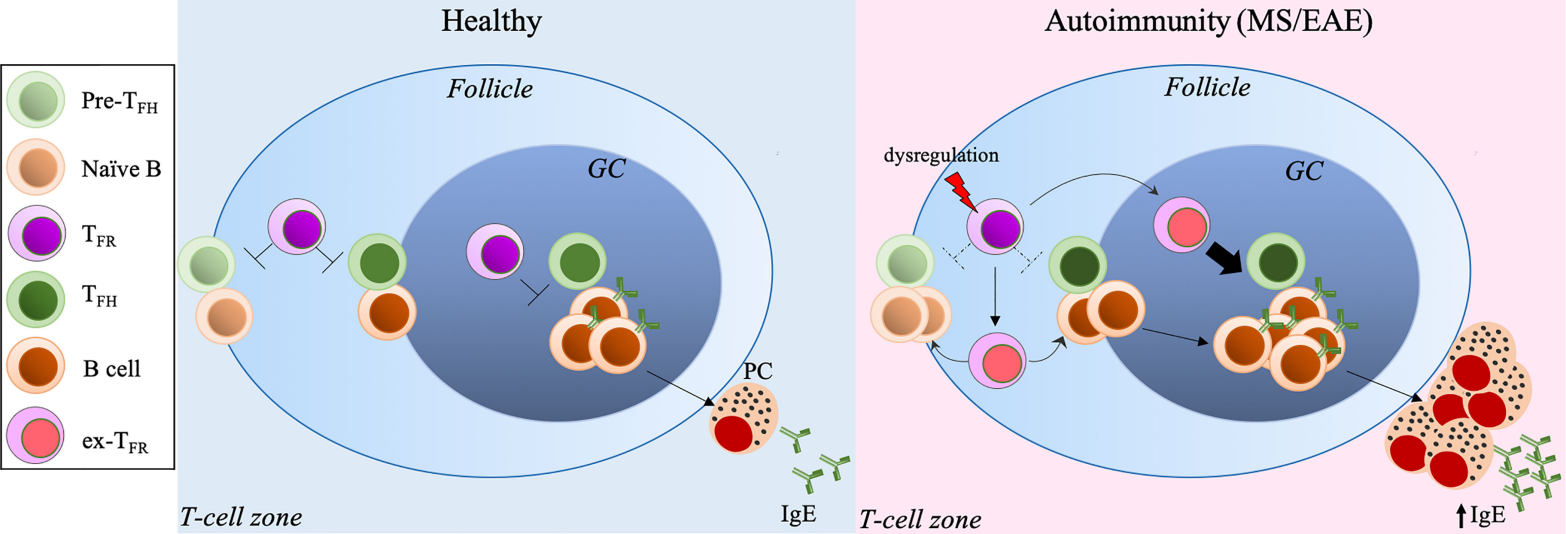
Excessive T_FH_-GC-B cell response and IgE production secondary to dysregulated T_FR_ cells exacerbate MS/EAE. Under normal conditions, IgE production is tightly controlled by the T_FH_-T_FR_ cellular pair. Dysregulation of T_FR_ cells leads to T_FH_/GC B-cell expansion and excessive IgE production, inducing autoreactive IgE, resulting in exacerbated autoimmune diseases, such as MS and EAE. PC, plasma cellse. x-T_FR_, desreguated T_FR_ cells.

Our recent studies have demonstrated that dysregulated T_FR_ cells result in aberrant T_FH_ cell expansion and excessive GC responses with increased antibody production (42). The stability and suppressive capacity of T_FR_ cells require the expression of the transcription factor, Blimp1. The deletion of Blimp1 in Foxp3^+^ Tregs reprograms Tregs and T_FR_ cells into effector T-cells that produce increased pro-inflammatory cytokines, such as IFNγ and IL-17A, promoting the autoimmune responses. Additionally, unstable T_FR_ cells migrate to the GC prematurely, thus promoting T_FH_ and GC B-cell expansion, along with upregulated antibody and autoantibody production (42) (**Figure 1**).

## Dysregulated B-Cells and Antibodies in CNS Autoimmunity

Dysregulation of B-cells directly or secondary to aberrant T-cell responses due to the immune tolerance breakdown can lead to autoantibody production, which is a classic hallmark of B-cell-mediated autoimmune diseases (46). Autoantibodies directed against membrane-associated, extracellular and nuclear antigens are found in a plethora of autoimmune disorders, including systemic lupus erythematosus (SLE), rheumatoid arthritis (RA) and type I diabetes (TID) (47–49), where they either directly injure target tissues or generate a polyclonal activation of the immune system *via* forming immune complexes and activating the complement cascade, resulting in unregulated inflammatory responses (46, 50–52). Conventionally, T_H_ cells, particularly T_H_1 and T_H_17 subsets, are thought to contribute heavily to MS and EAE (53). However, emerging evidence suggests that B-cells play a comparably crucial role in mediating disease through their ability to serve as cytokine-producing regulatory cells, professional APCs and ASCs (54). The impaired Breg activity may contribute to the development of MS and EAE, as mice deficient in Breg-derived IL-10 and IL-35 develop exacerbated EAE with failed recovery, while elevated IL-35^+^ or IL-10^+^ Bregs ameliorate disease severity (55, 56). Here we focus on the contribution of dysregulated T/B responses and resultant autoantibody production to MS and EAE.

Like other APCs, such as macrophages and dendritic cells, B-cells can present antigens to T-cells as an MHCII: peptide complex once antigens are recognized through their B-cell receptor (BCR) to activate T-cells, which in turn induce B-cell proliferation and differentiation into ASCs in SLOs. As expected, unchecked T/B response to self-antigens, like myelin-associated antigens, can promote CNS pathology. The expanded autoreactive B-cell clones are found in both the periphery, including draining cervical lymph nodes, and CNS compartments (meninges, CSF and parenchyma) of MS patients (57). In particular, IgG1^+^ B-cells expressing the chemokine receptor CXCR3 and the transcription factor T-bet are recruited and enriched in the CNS of some MS patients, likely reflecting high levels of CXCR3 ligand CXCL10 in the CSF of these patients (58, 59). These studies imply that autoreactive B-cells can traffic across the blood-brain barrier (BBB) into the CNS where they may undergo further differentiation into ASCs, promoting MS pathology (58, 60). Interestingly, histological analysis has revealed structures resembling lymphoid follicles compartmentalizing T, B, plasma cells, and follicular dendritic cells within the meninges of the CNS, termed as ectopic lymphoid structures (ELSs) (61). The presence of these follicle-like structures within the CNS further suggests that T/B-cell activation and proliferation *in situ* contribute to disease pathogenesis (62). T_FH_ and GC B-cells are found in these ELSs, but the presence of T_FR_ cells has not been reported in the inflamed CNS until our recent study (63), despite that the regulatory activity of T_FR_ cells in the lesions still require further investigation. Dysregulated T_FH_ cell-GC-antibody responses secondary to dysfunctional T_FR_ cells are the root of autoimmune disorders, including MS and EAE (64, 65) (**Figure 1**). MS patients have been found to harbor substantially reduced circulating T_FR_ cells compared to healthy controls, and those residual cells in the circulation resemble a more T_H_17-effector like phenotype with impaired suppressive activity (66). Our recent study has also shown that mice with dysfunctional T_FR_ cells due to the deletion of Blimp1 develop more severe EAE and fail to recover compared to control mice (63). In this MOG_35-55_-induced EAE model, Blimp1-deficient T_FR_ cells become unstable and produce T_H_17-associated cytokines, including GM-CSF and IL-17A. Moreover, these unstable T_FR_ cells acquire the T_FH_ helper activity, and promote GC B-cell expansion and antibody production, leading to augmented disease (63) (**Figure 1**).

Although the contribution of antibodies, particularly antibody isotypes, to the pathogenesis of MS and EAE remains largely unclear, antibodies specific to self-antigens, including myelin-derived lipids and proteins, are often detected in the CSF of MS patients (67–69). The emergence of oligoclonal bands (OCB) in the CSF, which are locally produced by clonally expanded antigen-experienced B-cells, is one of the hallmarks of MS, with more than 95% of MS patients containing the OCB of IgG class (8). RRMS patients with intrathecal IgG synthesis, manifested by the presence of OCB, are also associated with a higher risk of and shorter time to disability worsening (as reflected by the Expanded Disability Status Scale score) over a four-year period of follow-up (70). The antigen specificity of OCB remains elusive. Using an approach that allows concurrent analysis of the full-length sequences of matching Ig heavy and light chains from distinct OCB followed by producing recombinant OCB antibodies and antigen searches, Brändel et al. have revealed that six OCB antibodies from four MS patients recognize three ubiquitous intracellular proteins not specific to brain tissue (71). These interesting findings suggest that part of MS OCB antibodies target autoantigens released through tissue destruction (71). Additionally, Liu et al. have tested the specificity of recombinant IgG1 antibodies produced from clonally-expanded plasmablasts isolated from MS CSF. The application of these antibodies onto mouse organotypic cerebellar slices has shown that these antibodies bind to the surface of oligodendrocytes and myelinating axons inducing rapid demyelination accompanied with enhanced microglia activation in the presence of complement, suggesting their specificity to myelin-derived antigens (72). Another study has demonstrated that higher CSF IgM levels in treatment-naïve MS patients positively correlate with the CSF levels of molecules related to B-cell immunity (IL-10), recruitment (CXCL13 and CCL21), and macrophage/microglial activity (IL-12p70, CX3CL1 and CHI3L1), as well as white matter lesion numbers and disease activity after two years of follow-up, suggesting a potential role of IgM in the MS disease course (73). Several additional studies also implicate that intrathecal IgM synthesis may serve as a predictor of the onset of new relapses and worsened disease progression (74–77). In contrast, gut commensal-specific IgA-producing plasma cells have been shown to recirculate into the CNS of EAE mice and active MS patients during disease relapse (78, 79). Despite their unclear function in MS patients, these cells suppress neuroinflammation in EAE mice *via* the production of IL-10 (79), suggesting the anti-inflammatory role of IgA-producing cells which may serve as a predictor for MS disease course (78). Finally, the IgE isotype has only recently been implicated in taking part in MS pathology, as sera from some MS patients contain significantly higher levels of IgE reactive against myelin protein-derived peptides whereas peptide-reactive IgA or IgG is often undetectable (80). Our recent study has also pointed to a disease-promoting role of IgE in EAE (63). We will further discuss the pathogenic role of IgE in autoimmune diseases with an emphasis on MS and EAE in the following sections.

## Contribution of IgE to Autoimmune Diseases

### Regulation of IgE Production and Maintenance

B-cells undergo isotype switching, a complex process that requires precise and sequential genomic DNA splicing and recombination, to express surface membrane-bound IgE, and to secrete it as well. IgE is an antibody isotype with the lowest abundance and the shortest serum half-life *in vivo*, suggesting that IgE responses are generally limited at steady states (81). IgE can be derived from direct class-switching where IgM directly converts to IgE, or from sequential steps by initially converting IgM to IgG1 that then converts to IgE, or alternatively from IgG1 directly (81). These differing mechanisms for IgE generation are suggested to distinguish the IgE^+^ B-cell fates. While direct class-switching is associated with the generation of IgE^+^ GC B-cells and the production of low-affinity IgE, the sequential generation of IgE is more related to developing plasma cells with a greater affinity of IgE (82–85). Interestingly, the membrane-bound IgE (i.e., IgE^+^ BCR) has been shown *in vivo* and *in vitro* to autonomously promote B-cell differentiation into antibody-secreting plasma cells in the absence of cognate antigen, suggesting that BCR signals restrict IgE^+^ GC B-cell responses (86). Upon release into circulation, IgE binds to the high-affinity FcϵRI receptor expressed on the surface of mast cells, basophils and other cell types (87). Binding of IgE to FcϵRI expressed on dendritic cells and macrophages induces activation through the internalization of IgE-bound antigens, and production of a cytokine milieu (e.g., IL-4) favoring T_H_2 differentiation (81), which may in turn facilitate IgE class-switching, implying a positive feedback mechanism in the regulation of IgE production.

The IgE production is also tightly regulated by the T_FH_-T_FR_ cellular pair (**Figure 1**). T_FH_ cells have been shown to undergo progressive maturation after entering the GC to regulate GC B-cell differentiation, starting with an IL-21^+^ T_FH_ cells that enable the selection of high-affinity antibodies to become IL-4^+^ T_FH_ cells that promote the differentiation of ASCs, including IgE-producing cells (88). Although T_FR_ cells can positively induce IgE under certain settings (89, 90), we and others have reported that T_FR_ cells, particularly Blimp1^+^ T_FR_ cells, are key suppressors of IgE production (42, 63, 91–95). Using a unique T_FR_ cell-deleter mouse strain, T_FR_ cells have been demonstrated to control IgG and IgE responses and regulate IL-13-producing T_FH_ cell-induced IgE specifically in house dust mite models (94). A most recent study by Dr. Carola Vinuesa’s group has further shown that T_FR_ cells produce neuritin -a neuropeptide known to regulate synaptic plasticity and neuronal growth, migration, and survival- which regulates GC B-cells to block the emergence of autoantibodies and excessive IgE (95). The deletion of neuritin in Tregs or ablation of T_FR_ cells expedites the differentiation of GC B-cells into plasma cells and IgE class-switching (95). Thus, the immune system has adopted various mechanisms to restrain IgE production and keep it at proper levels under normal conditions.

### Unconventional Role of IgE in Autoimmune Disorders

Conventionally, IgE is known for mediating immediate, allergen-specific reactions, commonly referred to as type 1 hypersensitivity. Effector functions are triggered by the cross-linking of allergen-specific IgE to the FcϵRI receptor on mast cells and basophils, inducing the subsequent degranulation and release of potent proinflammatory mediators, such as histamine and T_H_2 cytokines, to cause inflammatory responses (96). IgE also binds to low-affinity receptors known as FcϵRII or CD23 on B-cells and other cells to mediate the uptake of IgE-antigen complexes (81, 97). Additionally, IgE plays an important evolutionary role in host protection against venoms and parasitic infections (98), particularly those caused by helminths and specific protozoans (99).

The association of elevated serum IgE levels with autoimmunity has been understudied, but recent clinical evidence linking autoreactive IgE beyond its conventional role in inducing allergic responses to autoimmune diseases has attracted more attention from the research community (100, 101). IgE has been implicated in contributing to the onset of inflammatory and autoimmune diseases such as RA, SLE, TID, atopic dermatitis, bullous pemphigoid, atherosclerosis, obesity, and coronary heart disease (102–106). Autoreactive IgE, including antinuclear IgE antibodies, are found in a significant portion of these patient populations (103, 104), despite that the exact pathogenic role of IgE remains largely unclear, and in some cases, autoreactive IgE levels are comparable to autoreactive IgG levels making a definitive causal relationship challenging to establish (100, 107). Notably, a key study of SLE patients demonstrates that double-stranded DNA (dsDNA)-reactive IgE can trigger IFNα production *via* activating plasmacytoid dendritic cells (pDCs) upon binding to FcϵRI on these cells, and that the serum concentrations of these dsDNA-reactive IgE correlate with disease severity (108). These studies have justified and stimulated the application of IgE blocker (omalizumab), which is used to treat allergic asthma, as a therapeutic option for some autoimmune disorders (109).

**Figure 2:**
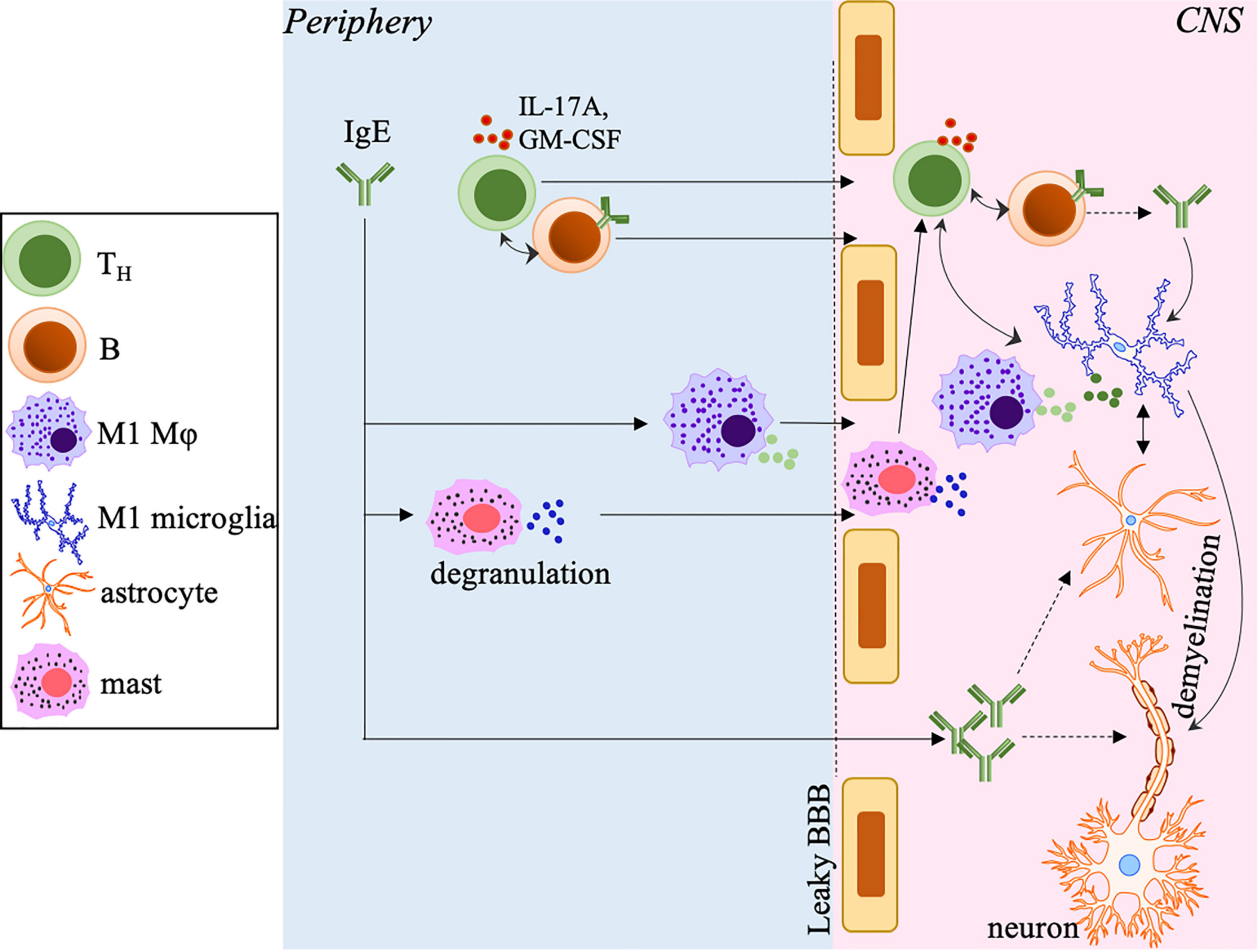
The potential pathogenic role of IgE in MS and EAE. In the periphery, IgE, including myelin-specific or autoreactive IgE, polarizes macrophages toward the M1 phenotype and induces mast cell degranulation, which promotes a leaky BBB allowing increased infiltration of T/B-cells, macrophages and mast cells into the CNS. IgE may also penetrate the BBB allowing the deposition into the CNS. In the CNS, IgE activates and polarizes microglia into the M1 phenotype. The M1 microglia and infiltrated macrophages produce inflammatory mediators to further drive the differentiation of encephalitogenic T_H_ cells, which in turn amplify the M1 polarization of these cells. IgE may directly activate astrocytes that cooperate with microglia to enhance neuroinflammation and induce axonal demyelination and destruction. IgE may also potentially directly target oligodendrocytes (not depicted) to promote axonal damage. The accumulation of T_H_ cells and B-cells may form ELSs that perpetuate inflammation, demyelination and disease progression. The small circles with different colors close to different cell types represent different secreted inflammatory mediators.

Supporting evidences from clinical and experimental data suggest the involvement of IgE in both MS and EAE (63, 80, 110). The discovery of significant serum levels of IgE specific to a panel of short, unique myelin protein-derived peptides in the cohort of 26 MS patients irrespective of clinical subtype based on an author-developed radioimmunoassay suggests that autoreactive IgE against CNS target antigens may hold valuable diagnostic potential in some MS patients (80). Indeed, IgE^+^ cells are detectable in all lesions of MS brains from 14 different patients, including areas with active immune infiltration (110). These studies also point out that the encephalitogenic potential of IgE may rely on its abundance in the CNS, and the presence of other pathogenic Ig isotypes, as they may compete with IgE for the same targets. Consistent with the findings that mice with disrupted Ig Fc receptors, including FcϵRI, display ameliorated EAE (111), we have recently revealed the increased CNS deposition of total and MOG-specific IgE in MOG_35-55_-induced EAE mice with dysregulated Tregs and T_FR_ cells, and the serum IgE levels positively correlate with EAE disease severity (63). The increased IL-17A and IL-4 produced by over-activated T_H_ cells and unstable Tregs in these mice may account for the elevated IgE (63), although other mechanisms, such as reduced neuritin expression by dysregulated T_FR_ cells, may be at play and are worth further investigation. Additionally, MOG-IgE (specific to the model antigen MOG_35-55_) but not MOG-IgG are elevated in these mice. Although MOG-IgG has become a new diagnostic marker for neuromyelitis optica (NMO) but not MS (112), we do not know if MOG-IgE is derived from MOG-IgG *via* class-switching in this pre-clinical study and if MOG-IgE may potentially contribute to NMO or MS. It will also be interesting to investigate if the IgE-mediated pathogenesis in other autoimmune disorders, such as activation of pDCs and production of IFNα, contributes to MS and EAE, despite that the role of type 1 IFNs in MS has been a contentious subject (113, 114). As one of the conventional target cells of IgE, earlier studies have suggested that mast cells in the CNS as a direct result of intravascular myelin-reactive IgE penetrating the BBB promote MS and EAE (80, 111, 115, 116). Our recent study has also suggested that CNS myeloid cells, including microglia and infiltrating macrophages, are targeted by IgE to induce their activation (63) (**Figure 2**). We will provide a more detailed discussion of this topic in the following section.

### IgE-Mediated Regulation of Microglia and Macrophages in MS and EAE

The crosstalk between the innate and adaptive arms of the immune system often occurs to ensure proper immune responses. Disruption within this complex network of cell communication can result in devastating consequences. In addition to T/B-cells and myeloid cells/macrophages that infiltrate into the CNS during MS and EAE progression, microglia, non-circulating tissue resident macrophages of the CNS, are critical for the regulation of neuroinflammation. Microglia are the cell types responsible for removing and pruning damaged and unnecessary neurons and synapses to maintain CNS homeostasis (117). Besides serving as APCs to induce T-cell activation, microglia and monocyte-derived macrophages are armed with several receptors equipped to remove aged, necrotic tissues and toxic molecules from their surroundings while eliciting endocytosis of immunocomplexes and complement-opsonized proteins through Fc receptors (118, 119). Upon homeostatic dysregulation, microglia and CNS-infiltrated macrophages can be pushed towards an M1 pro-inflammatory phenotype to mediate secretion of TNFα, IL-12, IL-6, IL-23, and TGFβ at elevated levels, promoting T_H_1 and T_H_17 differentiation, which in turn provide feedback to facilitate M1 polarization, and ultimately leading to MS pathology (120, 121). In MS and EAE, activated microglia and macrophages are hallmarks of active demyelinating lesions (122–124). Upon activation, microglia and macrophages can produce inflammatory cytokines, free radicals, protease and other mediators to augment CNS inflammation, destroy oligodendrocytes and cause axon injury and demyelination (125). The importance of microglia and macrophages in MS and EAE has been evidenced by the fact that the inhibition of microglial activation or downregulation of molecules in these cells to diminish their function results in disease attenuation along with reduced demyelination (125–127). However, it should be noted that microglia and macrophages can also provide neuroprotection by aiding in axonal remyelination and regeneration. Their beneficial and harmful effects are possibly attributed to the heterogeneity of these cells and temporal and spatial requirements during disease progression (125, 128, 129).

The impact of IgE on macrophages has been mainly investigated in atherosclerosis and cardiovascular diseases and more recently, in cancer (102, 130, 131). Using mice lacking IgE or FcϵRI combined with atherosclerosis prone models, IgE is reported to bind to FcϵRI on macrophages to induce activation, accumulation into the inflammatory lesions and skewed differentiation towards the pro-inflammatory M1 phenotype (102, 130, 131). Treatment of quiescent M0 and immunosuppressive M2 macrophages with IgE results in elevated levels of M1-associated molecules. In contrast, treatment of pro-inflammatory M1 cells retains the levels of these molecules, including CD80, TNFα, IFNγ and IL-1β (131), suggesting a favorable role of IgE in driving M1 type inflammatory responses. Our recent study has for the first time reported that IgE can activate CNS myeloid cells in the context of EAE (63). This study shows that supplementing sera from EAE mice into the CNS CD11b^+^ myeloid cell culture promotes activation and M1 polarization of these cells *via* upregulation of CD68, MHCII, and TNFα. Most notably, pre-incubation of sera with anti-IgE to neutralize IgE activity diminishes the activation of CD11b^+^ cells, suggesting a direct regulation of myeloid cells by IgE (63) (**Figure 2**), despite that a follow-up experiment is needed to distinguish the relative impact of IgE on microglia to CNS-infiltrated macrophages. The impact of IgE on other CNS resident cells, such as astrocytes, which may influence the disease course, is also warranted for future investigation. Moreover, further *in vivo* study and exploration of IgE’s mechanistic action are required to establish the importance of IgE in MS and EAE (**Figure 2**).

## Therapeutic Targeting B-Cells and Antibody Response to Treat MS and EAE

B-cell depletion therapy using monoclonal antibodies (mAbs) that target CD20 expressing B-cells is a highly effective treatment option for relapsing MS patients (132). Anti-CD20 mAbs work by depleting circulating CD20^+^ B-cells, with treatments lasting up to 9 months (133). There are currently three clinically approved anti-CD20 mAbs used in the treatment of MS patients: rituximab (RTX), ocrelizumab (OCR), and ofatumumab (OFA). These drugs differ in their clinical effectiveness based on their molecular structure and mechanism of action targeting B-cells, as reviewed elsewhere (132). The first of the three mAbs, RTX, a chimeric mouse-human mAb, was initially developed to target B-cell lymphomas, but is repurposed as an off-label treatment for MS patients and is now widely used in several autoimmune diseases (134–136). Its primary mechanism of action is complement-dependent cytotoxicity, but antibody-dependent cellular cytotoxicity (ADCC) also plays an important role in its effectiveness (137). OCR is used in patients with RRMS and PPMS. Currently, it is the only approved treatment for patients with the primary progressive form of the disease. OCR is a fully-humanized lytic mAb with ADCC as its primary mechanism of action; however apoptotic and antibody-dependent phagocytosis assist in B-cell depletion (138). OFA is a fully human mAb and is the only approved anti-CD20 mAb that uses a subcutaneous route of administration (139). Anti-CD20 clinical trials involving patients with RRMS show significant reductions in brain lesion formation, clinical relapse rates and no substantial side effects. However, a caveat is that CD20 is expressed on B-cells beginning in the late pre-B developmental stage, until the cells are terminally differentiated as antibody-secreting plasmablasts and plasma cells (140). Thus, pro-B cells and all ASCs are spared with anti-CD20 mAbs as they are CD20 negative (140), which may underlie the potential ineffectiveness, if any occurs, in some patients. The efficacy of B-cell depleting therapy may also need to consider the abundance and activity of Bregs, as transfer of these cells leads to disease mitigation, at least reported in EAE mice (141, 142). With the emergence of a critical pathogenic role of IgE in promoting EAE and MS, therapies against MS could be developed to neutralize IgE or block IgE activity, such as the use of omalizumab in other autoimmune disorders (109).

## Conclusions and Perspectives

Considerable progress has been made in uncovering the mechanisms guiding CNS autoimmunity, particularly in MS. However, there are many critical knowledge gaps that remain. It is essential to understand the emerging role of B-cells and IgE in triggering autoimmune diseases. Current and future areas of interest should include defining the effector activity of IgE in mediating macrophage/microglial activation, influencing the CNS resident cells and promoting a pro-inflammatory milieu in the CNS. Better delineation of autoreactivity of autoantibodies may enable immunological phenotyping of MS patients, facilitate the development of a diagnostic test and lead to new treatment directions. In addition, as our knowledge of B-cell involvement in MS progression, elucidating the mechanisms by which T_FR_, T_FH_, GC-B cells and antibody production (particularly IgE) are properly regulated is crucial in discovering disease-promoting factors. This represents a new era for identifying therapeutic targets to manipulate B-cell activity for targeted immunotherapies.

## Author Contributions

MRS, MMM, JDL, and JWL drafted the manuscript, revised it critically, and approved this final version for publication.

## Conflict of Interest

The authors declare that the research was conducted in the absence of any commercial or financial relationships that could be construed as a potential conflict of interest.

## Publisher’s Note

All claims expressed in this article are solely those of the authors and do not necessarily represent those of their affiliated organizations, or those of the publisher, the editors and the reviewers. Any product that may be evaluated in this article, or claim that may be made by its manufacturer, is not guaranteed or endorsed by the publisher.

## References

[1] Walton C King R Rechtman L Kaye W Leray E Marrie RA. Rising Prevalence of Multiple Sclerosis Worldwide: Insights From the Atlas of MS, Third Edition. Mult Scler (2020) 26(14):1816–21. doi: 10.1177/1352458520970841 PMC7720355 33174475

[2] Reich DS Lucchinetti CF Calabresi PA. Multiple Sclerosis. N Engl J Med (2018) 378(2):169–80. doi: 10.1056/NEJMra1401483 PMC6942519 29320652

[3] Ruiz F Vigne S Pot C. Resolution of Inflammation During Multiple Sclerosis. Semin Immunopathol (2019) 41(6):711–26. doi: 10.1007/s00281-019-00765-0 PMC6881249 31732775

[4] Sospedra M Martin R. Immunology of Multiple Sclerosis. Annu Rev Immunol (2005) 23:683–747. doi: 10.1146/annurev.immunol.23.021704.115707 15771584

[5] Lassmann H Bradl M. Multiple Sclerosis: Experimental Models and Reality. Acta Neuropathol (2017) 133(2):223–44. doi: 10.1007/s00401-016-1631-4 PMC5250666 27766432

[6] Dendrou CA Fugger L Friese MA. Immunopathology of Multiple Sclerosis. Nat Rev Immunol (2015) 15:13. doi: 10.1038/nri3871 26250739

[7] Cross AH Trotter JL Lyons J. B Cells and Antibodies in CNS Demyelinating Disease. J Neuroimmunol (2001) 112(1-2):1–14. doi: 10.1016/s0165-5728(00)00409-4 11108928

[8] Link H Huang YM. Oligoclonal Bands in Multiple Sclerosis Cerebrospinal Fluid: An Update on Methodology and Clinical Usefulness. J Neuroimmunol (2006) 180(1-2):17–28. doi: 10.1016/j.jneuroim.2006.07.006 16945427

[9] Cross AH Klein RS Piccio L. Rituximab Combination Therapy in Relapsing Multiple Sclerosis. Ther Adv Neurol Disord (2012) 5(6):311–9. doi: 10.1177/1756285612461165 PMC3487534 23139702

[10] Hauser SL Waubant E Arnold DL Vollmer T Antel J Fox RJ. B-Cell Depletion With Rituximab in Relapsing-Remitting Multiple Sclerosis. N Engl J Med (2008) 358(7):676–88. doi: 10.1056/NEJMoa0706383 18272891

[11] Hauser SL Belachew S Kappos L. Ocrelizumab in Primary Progressive and Relapsing Multiple Sclerosis. N Engl J Med (2017) 376(17):1694. doi: 10.1056/NEJMc1702076 28445663

[12] Hauser SL Bar-Or A Cohen JA Comi G Correale J Coyle PK. Ofatumumab Versus Teriflunomide in Multiple Sclerosis. N Engl J Med (2020) 383(6):546–57. doi: 10.1056/NEJMoa1917246 32757523

[13] Ollila J Vihinen M. B Cells. Int J Biochem Cell Biol (2005) 37(3):518–23. doi: 10.1016/j.biocel.2004.09.007 15618007

[14] Eibel H Kraus H Sic H Kienzler AK Rizzi M. B Cell Biology: An Overview. Curr Allergy Asthma Rep (2014) 14(5):434. doi: 10.1007/s11882-014-0434-8 24633618

[15] Jankovic M Casellas R Yannoutsos N Wardemann H Nussenzweig MC. RAGs and Regulation of Autoantibodies. Annu Rev Immunol (2004) 22:485–501. doi: 10.1146/annurev.immunol.22.012703.104707 15032586

[16] Zhou Y Zhang Y Han J Yang M Zhu J Jin T. Transitional B Cells Involved in Autoimmunity and Their Impact on Neuroimmunological Diseases. J Transl Med (2020) 18(1):131. doi: 10.1186/s12967-020-02289-w 32183811 PMC7079408

[17] Tellier J Nutt SL. Plasma Cells: The Programming of an Antibody-Secreting Machine. Eur J Immunol (2019) 49(1):30–7. doi: 10.1002/eji.201847517 30273443

[18] Akkaya M Kwak K Pierce SK. B Cell Memory: Building Two Walls of Protection Against Pathogens. Nat Rev Immunol (2020) 20(4):229–38. doi: 10.1038/s41577-019-0244-2 PMC7223087 31836872

[19] Hattori T Lai D Dementieva IS Montano SP Kurosawa K Zheng Y. Antigen Clasping by Two Antigen-Binding Sites of an Exceptionally Specific Antibody for Histone Methylation. Proc Natl Acad Sci U S A (2016) 113(8):2092–7. doi: 10.1073/pnas.1522691113 PMC4776465 26862167

[20] Forthal DN. Functions of Antibodies. Microbiol Spectr (2014) 2(4):1–17. doi: 10.1128/microbiolspec.AID-0019-2014 PMC4159104 25215264

[21] Chen X Jensen PE. The Role of B Lymphocytes as Antigen-Presenting Cells. Arch Immunol Ther Exp (Warsz) (2008) 56(2):77–83. doi: 10.1007/s00005-008-0014-5 18373241

[22] Shen P Fillatreau S. Antibody-Independent Functions of B Cells: A Focus on Cytokines. Nat Rev Immunol (2015) 15(7):441–51. doi: 10.1038/nri3857 26065586

[23] Vazquez MI Catalan-Dibene J Zlotnik A. B Cells Responses and Cytokine Production are Regulated by Their Immune Microenvironment. Cytokine (2015) 74(2):318–26. doi: 10.1016/j.cyto.2015.02.007 PMC4475485 25742773

[24] Lund FE. Cytokine-Producing B Lymphocytes-Key Regulators of Immunity. Curr Opin Immunol (2008) 20(3):332–8. doi: 10.1016/j.coi.2008.03.003 PMC2474694 18417336

[25] Knier B Hiltensperger M Sie C Aly L Lepennetier G Engleitner T. Myeloid-Derived Suppressor Cells Control B Cell Accumulation in the Central Nervous System During Autoimmunity. Nat Immunol (2018) 19(12):1341–51. doi: 10.1038/s41590-018-0237-5 PMC6241855 30374128

[26] Rosser EC Mauri C. Regulatory B Cells: Origin, Phenotype, and Function. Immunity (2015) 42(4):607–12. doi: 10.1016/j.immuni.2015.04.005 25902480

[27] Sokolov AV Shmidt AA. Lomakin Ya. B Cell Regul Autoimmune Diseases Acta Naturae (2018) 10(3):11–22. doi: 10.32607/20758251-2018-10-3-11-22 PMC6209408 30397522

[28] Wang RX Yu CR Dambuza IM Mahdi RM Dolinska MB Sergeev YV. Interleukin-35 Induces Regulatory B Cells That Suppress Autoimmune Disease. Nat Med (2014) 20(6):633–41. doi: 10.1038/nm.3554 PMC4048323 24743305

[29] Bosma A Abdel-Gadir A Isenberg DA Jury EC Mauri C. Lipid-Antigen Presentation by CD1d(+) B Cells is Essential for the Maintenance of Invariant Natural Killer T Cells. Immunity (2012) 36(3):477–90. doi: 10.1016/j.immuni.2012.02.008 PMC3391684 22406267

[30] Nguyen DC Joyner CJ Sanz I Lee FE. Factors Affecting Early Antibody Secreting Cell Maturation Into Long-Lived Plasma Cells. Front Immunol (2019) 10:2138. doi: 10.3389/fimmu.2019.02138 31572364 PMC6749102

[31] Stebegg M Kumar SD Silva-Cayetano A Fonseca VR Linterman MA Graca L. Regulation of the Germinal Center Response. Front Immunol (2018) 9:2469. doi: 10.3389/fimmu.2018.02469 30410492 PMC6209676

[32] Martin A Chahwan R Parsa JY Scharff MD. Somatic Hypermutation: The Molecular Mechanism Underlying the Production of Effective High-Affinity Antibodies. In: Tasuku Honjo MR Radbruch A Alt F, editors. Molecular Biology of B Cells, 2nd ed. Cambridge, MA: Academic Press (2014). p. 363–88.

[33] Leavenworth JW Verbinnen B Yin J Huang H Cantor H. A P85alpha-Osteopontin Axis Couples the Receptor ICOS to Sustained Bcl-6 Expression by Follicular Helper and Regulatory T Cells. Nat Immunol (2015) 16(1):96–106. doi: 10.1038/ni.3050 25436971 PMC4405167

[34] Shen E Wang Q Rabe H Liu W Cantor H Leavenworth JW. Chromatin Remodeling by the NuRD Complex Regulates Development of Follicular Helper and Regulatory T Cells. Proc Natl Acad Sci U S A (2018) 115(26):6780–5. doi: 10.1073/pnas.1805239115 PMC6042103 29891681

[35] Johnston RJ Poholek AC DiToro D Yusuf I Eto D Barnett B. Bcl6 and Blimp-1 are Reciprocal and Antagonistic Regulators of T Follicular Helper Cell Differentiation. Science (2009) 325(5943):1006–10. doi: 10.1126/science.1175870 PMC2766560 19608860

[36] Nurieva RI Chung Y Martinez GJ Yang XO Tanaka S Matskevitch TD. Bcl6 Mediates the Development of T Follicular Helper Cells. Science (2009) 325(5943):1001–5. doi: 10.1126/science.1176676 PMC2857334 19628815

[37] Linterman MA Beaton L Yu D Ramiscal RR Srivastava M Hogan JJ. IL-21 Acts Directly on B Cells to Regulate Bcl-6 Expression and Germinal Center Responses. J Exp Med (2010) 207(2):353–63. doi: 10.1084/jem.20091738 PMC2822609 20142429

[38] Finkin S Hartweger H Oliveira TY Kara EE Nussenzweig MC. Protein Amounts of the MYC Transcription Factor Determine Germinal Center B Cell Division Capacity. Immunity (2019) 51(2):12. doi: 10.1016/j.immuni.2019.06.013 31350178 PMC6703930

[39] Mintz MA Cyster JG. T Follicular Helper Cells in Germinal Center B Cell Selection and Lymphomagenesis. Immunol Rev (2020) 296(1):48–61. doi: 10.1111/imr.12860 32412663 PMC7817257

[40] Vinuesa CG Tangye SG Moser B Mackay CR. Follicular B Helper T Cells in Antibody Responses and Autoimmunity. Nat Rev Immunol (2005) 5:12. doi: 10.1038/nri1714 16261173

[41] Vinuesa CG MC Angelucci C Athanasopoulos V Rui L Hill KM Yu D. A RING-Type Ubiquitin Ligase Family Member Required to Repress Follicular Helper T Cells and Autoimmunity. Nature (2005) 435:6. doi: 10.1038/nature03555 15917799

[42] Shen E Rabe H Luo L Wang L Wang Q Yin J. Control of Germinal Center Localization and Lineage Stability of Follicular Regulatory T Cells by the Blimp1 Transcription Factor. Cell Rep (2019) 29(7):1848–61.e6. doi: 10.1016/j.celrep.2019.10.012 31722202 PMC6897316

[43] Linterman MA Pierson W Lee SK Kallies A Kawamoto S Rayner TF. Foxp3+ Follicular Regulatory T Cells Control the Germinal Center Response. Nat Med (2011) 17(8):975–82. doi: 10.1038/nm.2425 PMC3182542 21785433

[44] Wollenberg I Agua-Doce A Hernandez A Almeida C Oliveira VG Faro J. Regulation of the Germinal Center Reaction by Foxp3+ Follicular Regulatory T Cells. J Immunol (2011) 187(9):4553–60. doi: 10.4049/jimmunol.1101328 21984700

[45] Chung Y Tanaka S Chu F Nurieva RI Martinez GJ Rawal S. Follicular Regulatory T Cells Expressing Foxp3 and Bcl-6 Suppress Germinal Center Reactions. Nat Med (2011) 17(8):983–8. doi: 10.1038/nm.2426 PMC3151340 21785430

[46] Elkon K Casali P. Nature and Functions of Autoantibodies. Nat Clin Pract Rheumatol (2008) 4(9):491–8. doi: 10.1038/ncprheum0895 PMC2703183 18756274

[47] Bingley PJ. Clinical Applications of Diabetes Antibody Testing. J Clin Endocrinol Metab (2010) 95(1):25–33. doi: 10.1210/jc.2009-1365 19875480

[48] Hampe CS. B Cell in Autoimmune Diseases. Scientifica (Cairo) (2012) 2012:215308. doi: 10.6064/2012/215308 PMC3692299 23807906

[49] Martin F Chan AC. Pathogenic Roles of B Cells in Human Autoimmunity; Insights From the Clinic. Immunity (2004) 20(5):517–27. doi: 10.1016/s1074-7613(04)00112-8 15142521

[50] Racanelli V Prete M Musaraj G Dammacco F Perosa F. Autoantibodies to Intracellular Antigens: Generation and Pathogenetic Role. Autoimmun Rev (2011) 10(8):503–8. doi: 10.1016/j.autrev.2011.03.001 21397735

[51] Ballanti E Perricone C Greco E Ballanti M Di Muzio G Chimenti MS. Complement and Autoimmunity. Immunol Res (2013) 56(2-3):477–91. doi: 10.1007/s12026-013-8422-y 23615835

[52] Chen M Daha MR Kallenberg CG. The Complement System in Systemic Autoimmune Disease. J Autoimmun (2010) 34(3):J276–86. doi: 10.1016/j.jaut.2009.11.014 20005073

[53] Lehmann-Horn K Kronsbein HC Weber MS. Targeting B Cells in the Treatment of Multiple Sclerosis: Recent Advances and Remaining Challenges. Ther Adv Neurol Disord (2013) 6(3):161–73. doi: 10.1177/1756285612474333 PMC3625013 23634189

[54] Jain RW Yong VW. B Cells in Central Nervous System Disease: Diversity, Locations and Pathophysiology. Nat Rev Immunol (2021) 13:1–12. doi: 10.1038/s41577-021-00652-6 PMC8667979 34903877

[55] Radomir L Kramer MP Perpinial M Schottlender N Rabani S David K. The Survival and Function of IL-10-Producing Regulatory B Cells are Negatively Controlled by SLAMF5. Nat Commun (2021) 12(1):1893. doi: 10.1038/s41467-021-22230-z 33767202 PMC7994628

[56] Egwuagu CE Yu CR. Interleukin 35-Producing B Cells (I35-Breg): A New Mediator of Regulatory B-Cell Functions in CNS Autoimmune Diseases. Crit Rev Immunol (2015) 35(1):49–57. doi: 10.1615/critrevimmunol.2015012558 25746047 PMC5433835

[57] Stern JN Yaari G Vander Heiden JA Church G Donahue WF Hintzen RQ. B Cells Populating the Multiple Sclerosis Brain Mature in the Draining Cervical Lymph Nodes. Sci Transl Med (2014) 6(248):248ra107. doi: 10.1126/scitranslmed.3008879 PMC4388137 25100741

[58] van Langelaar J Rijvers L Janssen M Wierenga-Wolf AF Melief MJ Siepman TA. Induction of Brain-Infiltrating T-Bet-Expressing B Cells in Multiple Sclerosis. Ann Neurol (2019) 86(2):264–78. doi: 10.1002/ana.25508 PMC6771938 31136008

[59] Sorensen TL Trebst C Kivisakk P Klaege KL Majmudar A Ravid R. Multiple Sclerosis: A Study of CXCL10 and CXCR3 Co-Localization in the Inflamed Central Nervous System. J Neuroimmunol (2002) 127(1-2):59–68. doi: 10.1016/s0165-5728(02)00097-8 12044976

[60] Palanichamy A Apeltsin L Kuo TC Sirota M Wang S Pitts SJ. Immunoglobulin Class-Switched B Cells Form an Active Immune Axis Between CNS and Periphery in Multiple Sclerosis. Sci Transl Med (2014) 6(248):248ra106. doi: 10.1126/scitranslmed.3008930 PMC4176763 25100740

[61] Serafini B Rosicarelli B Magliozzi R Stigliano E Aloisi F. Detection of Ectopic B-Cell Follicles With Germinal Centers in the Meninges of Patients With Secondary Progressive Multiple Sclerosis. Brain Pathol (2004) 14(2):164–74. doi: 10.1111/j.1750-3639.2004.tb00049.x PMC8095922 15193029

[62] Magliozzi R Howell O Vora A Serafini B Nicholas R Puopolo M. Meningeal B-Cell Follicles in Secondary Progressive Multiple Sclerosis Associate With Early Onset of Disease and Severe Cortical Pathology. Brain (2007) 130(Pt 4):1089–104. doi: 10.1093/brain/awm038 17438020

[63] Luo L Hu X Dixon ML Pope BJ Leavenworth JD Raman C. Dysregulated Follicular Regulatory T Cells and Antibody Responses Exacerbate Experimental Autoimmune Encephalomyelitis. J Neuroinflamm (2021) 18(1):27. doi: 10.1186/s12974-021-02076-4 PMC7814531 33468194

[64] Crotty S. T Follicular Helper Cell Differentiation, Function, and Roles in Disease. Immunity (2014) 41(4):529–42. doi: 10.1016/j.immuni.2014.10.004 PMC4223692 25367570

[65] Quinn JL Kumar G Agasing A Ko RM Axtell RC. Role of TFH Cells in Promoting T Helper 17-Induced Neuroinflammation. Front Immunol (2018) 9:382. doi: 10.3389/fimmu.2018.00382 29535739 PMC5835081

[66] Dhaeze T Peelen E Hombrouck A Peeters L Van Wijmeersch B Lemkens N. Circulating Follicular Regulatory T Cells Are Defective in Multiple Sclerosis. J Immunol (2015) 195(3):832–40. doi: 10.4049/jimmunol.1500759 26071562

[67] Fitzner B Hecker M Zettl UK. Molecular Biomarkers in Cerebrospinal Fluid of Multiple Sclerosis Patients. Autoimmun Rev (2015) 14(10):903–13. doi: 10.1016/j.autrev.2015.06.001 26071103

[68] Warren KG Catz I. Relative Frequency of Autoantibodies to Myelin Basic Protein and Proteolipid Protein in Optic Neuritis and Multiple Sclerosis Cerebrospinal Fluid. J Neurol Sci (1994) 121(1):66–73. doi: 10.1016/0022-510x(94)90158-9 7510787

[69] Brennan KM Galban-Horcajo F Rinaldi S O'Leary CP Goodyear CS Kalna G. Lipid Arrays Identify Myelin-Derived Lipids and Lipid Complexes as Prominent Targets for Oligoclonal Band Antibodies in Multiple Sclerosis. J Neuroimmunol (2011) 238(1-2):87–95. doi: 10.1016/j.jneuroim.2011.08.002 21872346 PMC3400538

[70] Gasperi C Salmen A Antony G Bayas A Heesen C Kumpfel T. German Competence Network of Multiple S. Association of Intrathecal Immunoglobulin G Synthesis With Disability Worsening in Multiple Sclerosis. JAMA Neurol (2019) 76(7):841–9. doi: 10.1001/jamaneurol.2019.0905 PMC6583696 31034002

[71] Brandle SM Obermeier B Senel M Bruder J Mentele R Khademi M. Distinct Oligoclonal Band Antibodies in Multiple Sclerosis Recognize Ubiquitous Self-Proteins. Proc Natl Acad Sci U S A (2016) 113(28):7864–9. doi: 10.1073/pnas.1522730113 PMC4948369 27325759

[72] Liu Y Given KS Harlow DE Matschulat AM Macklin WB Bennett JL. Myelin-Specific Multiple Sclerosis Antibodies Cause Complement-Dependent Oligodendrocyte Loss and Demyelination. Acta Neuropathologica Commun (2017) 5(25):13. doi: 10.1186/s40478-017-0428-6 PMC5366134 28340598

[73] Magliozzi R Mazziotti V Montibeller L Pisani AI Marastoni D Tamanti A. Cerebrospinal Fluid IgM Levels in Association With Inflammatory Pathways in Multiple Sclerosis Patients. Front Cell Neurosci (2020) 14:569827. doi: 10.3389/fncel.2020.569827 33192314 PMC7596330

[74] Villar LM Masjuan J Gonzalez-Porque P Plaza J Sadaba MC Roldan E. Intrathecal IgM Synthesis Predicts the Onset of New Relapses and a Worse Disease Course in MS. Neurology (2002) 59(4):555–9. doi: 10.1212/wnl.59.4.555 12196648

[75] Perini P Ranzato F Calabrese M Battistin L Gallo P. Intrathecal IgM Production at Clinical Onset Correlates With a More Severe Disease Course in Multiple Sclerosis. J Neurol Neurosurg Psychiatry (2006) 77(8):953–5. doi: 10.1136/jnnp.2005.086116 PMC2077642 16574727

[76] Fonderico M Biagioli T Lanzilao L Bellinvia A Fratangelo R Pasto L. Prognostic Role of Intrathecal IgM Synthesis in Multiple Sclerosis: Results From a Clinical Series. Mult Scler (2021) 27(2):198–207. doi: 10.1177/1352458520907913 32091300

[77] Monreal E Sainz de la Maza S Costa-Frossard L Walo-Delgado P Zamora J Fernandez-Velasco JI. Predicting Aggressive Multiple Sclerosis With Intrathecal IgM Synthesis Among Patients With a Clinically Isolated Syndrome. Neurol Neuroimmunol Neuroinflamm (2021) 8(5):e1047. doi: 10.1212/NXI.0000000000001047 PMC8299514 34301819

[78] Probstel AK Zhou X Baumann R Wischnewski S Kutza M Rojas OL. Gut Microbiota-Specific IgA(+) B Cells Traffic to the CNS in Active Multiple Sclerosis. Sci Immunol (2020) 5(53):eabc7191. doi: 10.1126/sciimmunol.abc7191 PMC8043673 33219152

[79] Rojas OL Probstel AK Porfilio EA Wang AA Charabati M Sun T. Recirculating Intestinal IgA-Producing Cells Regulate Neuroinflammation *via* IL-10. Cell (2019) 176(3):610–24.e18. doi: 10.1016/j.cell.2018.11.035 30612739 PMC6903689

[80] Mikol DD Ditlow C Usatin D Biswas P Kalbfleisch J Milner A. Serum IgE Reactive Against Small Myelin Protein-Derived Peptides is Increased in Multiple Sclerosis Patients. J Neuroimmunol (2006) 180(1-2):40–9. doi: 10.1016/j.jneuroim.2006.06.030 16996143

[81] Wu LC Zarrin AA. The Production and Regulation of IgE by the Immune System. Nat Rev Immunol (2014) 14:12. doi: 10.1038/nri3632 24625841

[82] He JS Meyer-Hermann M Xiangying D Zuan LY Jones LA Ramakrishna L. The Distinctive Germinal Center Phase of IgE+ B Lymphocytes Limits Their Contribution to the Classical Memory Response. J Exp Med (2013) 210(12):2755–71. doi: 10.1084/jem.20131539 PMC3832920 24218137

[83] Xiong H Dolpady J Wabl M Curotto de Lafaille MA Lafaille JJ. Sequential Class Switching is Required for the Generation of High Affinity IgE Antibodies. J Exp Med (2012) 209(2):353–64. doi: 10.1084/jem.20111941 PMC3280879 22249450

[84] Zhang T Franklin A Boboila C McQuay A Gallagher MP Manis JP. Downstream Class Switching Leads to IgE Antibody Production by B Lymphocytes Lacking IgM Switch Regions. Proc Natl Acad Sci U S A (2010) 107(7):3040–5. doi: 10.1073/pnas.0915072107 PMC2840363 20133637

[85] Jung S Siebenkotten G Radbruch A. Frequency of Immunoglobulin E Class Switching is Autonomously Determined and Independent of Prior Switching to Other Classes. J Exp Med (1994) 179(6):2023–6. doi: 10.1084/jem.179.6.2023 PMC2191511 8195724

[86] Yang Z Robinson MJ Chen X Smith GA Taunton J Liu W. Regulation of B Cell Fate by Chronic Activity of the IgE B Cell Receptor. eLife (2016) 5:31. doi: 10.7554/eLife.21238 PMC5207771 27935477

[87] Kelly BT Grayson MH. What is it Good for? Ann Allergy Asthma Immunol (2016) 116(3):183–7. doi: 10.1016/j.anai.2015.10.026 PMC4784105 26945494

[88] Weinstein JS Herman EI Lainez B Licona-Limon P Esplugues E Flavell R. TFH Cells Progressively Differentiate to Regulate the Germinal Center Response. Nat Immunol (2016) 17(10):1197–205. doi: 10.1038/ni.3554 PMC5030190 27573866

[89] Koh B Ulrich BJ Nelson AS Panangipalli G Kharwadkar R Wu W. Bcl6 and Blimp1 Reciprocally Regulate ST2(+) Treg-Cell Development in the Context of Allergic Airway Inflammation. J Allergy Clin Immunol (2020) 146(5):1121–36.e9. doi: 10.1016/j.jaci.2020.03.002 32179158 PMC7487006

[90] Xie MM Chen Q Liu H Yang K Koh B Wu H. T Follicular Regulatory Cells and IL-10 Promote Food Antigen-Specific IgE. J Clin Invest (2020) 130(7):3820–32. doi: 10.1172/JCI132249 PMC7324176 32255767

[91] Dixon ML Leavenworth JD Leavenworth JW. Lineage Reprogramming of Effector Regulatory T Cells in Cancer. Front Immunol (2021) 12:717421. doi: 10.3389/fimmu.2021.717421 34394124 PMC8355732

[92] Dixon ML Luo L Ghosh S Grimes JM Leavenworth JD Leavenworth JW. Remodeling of the Tumor Microenvironment *via* Disrupting Blimp1(+) Effector Treg Activity Augments Response to Anti-PD-1 Blockade. Mol Cancer (2021) 20(1):150. doi: 10.1186/s12943-021-01450-3 34798898 PMC8605582

[93] Leavenworth JW Shi LZ Wang X Wei H. Editorial: Immune Cell Lineage Reprogramming in Cancer. Front Immunol (2021) 12:838464. doi: 10.3389/fimmu.2021.838464 35126385 PMC8807485

[94] Clement RL Daccache J Mohammed MT Diallo A Blazar BR Kuchroo VK. Follicular Regulatory T Cells Control Humoral and Allergic Immunity by Restraining Early B Cell Responses. Nat Immunol (2019) 20(10):1360–71. doi: 10.1038/s41590-019-0472-4 PMC6754271 31477921

[95] Gonzalez-Figueroa JAR I Villacís LN Stanley M Linterman MA Dent A Canete PF. Follicular Regulatory T Cells Produce Neuritin to Regulate B Cells. Cell (2021) 184:14. doi: 10.1016/j.cell.2021.02.027 33711260

[96] Sutton BJ Davies AM Bax HJ Karagiannis SN. IgE Antibodies: From Structure to Function and Clinical Translation. Antibodies (Basel) (2019) 8(1):19. doi: 10.3390/antib8010019 PMC6640697 31544825

[97] Presta L Shields R O'Connell L Lahr S Porter J Gorman C. The Binding Site on Human Immunoglobulin E for its High Affinity Receptor. J Biol Chem (1994) 269(42):26368–73. doi: 10.1016/S0021-9258(18)47203-1 7929356

[98] Mukai K Tsai M Starkl P Marichal T Galli SJ. IgE and Mast Cells in Host Defense Against Parasites and Venoms. Semin Immunopathol (2016) 38(5):581–603. doi: 10.1007/s00281-016-0565-1 27225312 PMC5010491

[99] Lynch NR Hagel IA Palenque ME Di Prisco MC Escudero JE Corao LA. Relationship Between Helminthic Infection and IgE Response in Atopic and Nonatopic Children in a Tropical Environment. J Allergy Clin Immunol (1998) 101(2 Pt 1):217–21. doi: 10.1016/S0091-6749(98)70386-0 9500755

[100] Ettinger R Karnell JL Henault J Panda SK Riggs JM Kolbeck R. Pathogenic Mechanisms of IgE-Mediated Inflammation in Self-Destructive Autoimmune Responses. Autoimmunity (2017) 50(1):25–36. doi: 10.1080/08916934.2017.1280670 28166684

[101] Tsokos GC. Autoimmunity and Organ Damage in Systemic Lupus Erythematosus. Nat Immunol (2020) 21(6):605–14. doi: 10.1038/s41590-020-0677-6 PMC8135909 32367037

[102] Zhang X Li J Luo S Wang M Huang Q Deng Z. IgE Contributes to Atherosclerosis and Obesity by Affecting Macrophage Polarization, Macrophage Protein Network, and Foam Cell Formation. Arterioscler Thromb Vasc Biol (2020) 40(3):597–610. doi: 10.1161/ATVBAHA.119.313744 31996021 PMC7047522

[103] Permin H Wiik A. The Prevalence of IgE Antinuclear Antibodies in Rheumatoid Arthritis and Systemic Lupus Erythematosus. Acta Pathol Microbiol Scand C (1978) 86C(5):245–9. doi: 10.1111/j.1699-0463.1978.tb02587.x 309705

[104] Kopecki Z Stevens NE Chong HT Yang GN Cowin AJ. Flightless I Alters the Inflammatory Response and Autoantibody Profile in an OVA-Induced Atopic Dermatitis Skin-Like Disease. Front Immunol (2018) 9:1833. doi: 10.3389/fimmu.2018.01833 30147695 PMC6095979

[105] Wang Z Zhang H Shen XH Jin KL Ye GF Qiu W. Immunoglobulin E and Mast Cell Proteases are Potential Risk Factors of Impaired Fasting Glucose and Impaired Glucose Tolerance in Humans. Ann Med (2013) 45(3):220–9. doi: 10.3109/07853890.2012.732234 PMC3934348 23110545

[106] Wang J Cheng X Xiang MX Alanne-Kinnunen M Wang JA Chen H. IgE Stimulates Human and Mouse Arterial Cell Apoptosis and Cytokine Expression and Promotes Atherogenesis in Apoe-/- Mice. J Clin Invest (2011) 121(9):3564–77. doi: 10.1172/JCI46028 PMC3163955 21821913

[107] Maurer M Altrichter S Schmetzer O Scheffel J Church MK Metz M. Immunoglobulin E-Mediated Autoimmunity. Front Immunol (2018) 9:689. doi: 10.3389/fimmu.2018.00689 29686678 PMC5900004

[108] Henault J Riggs JM Karnell JL Liarski VM Li J Shirinian L. Self-Reactive IgE Exacerbates Interferon Responses Associated With Autoimmunity. Nat Immunol (2016) 17(2):196–203. doi: 10.1038/ni.3326 26692173 PMC4718782

[109] Sanjuan MA Sagar D Kolbeck R. Role of IgE in Autoimmunity. J Allergy Clin Immunol (2016) 137(6):1651–61. doi: 10.1016/j.jaci.2016.04.007 27264000

[110] Toms R Weiner HL Johnson D. Identification of IgE-Positive Cells and Mast Cells in Frozen Sections of Multiple Sclerosis Brains. J Neuroimmunol (1990) 30(2-3):169–77. doi: 10.1016/0165-5728(90)90101-r 2229408

[111] Pedotti R DeVoss JJ Youssef S Mitchell D Wedemeyer J Madanat R. Multiple Elements of the Allergic Arm of the Immune Response Modulate Autoimmune Demyelination. Proc Natl Acad Sci U S A (2003) 100(4):1867–72. doi: 10.1073/pnas.252777399 PMC149925 12576552

[112] Jarius S Ruprecht K Kleiter I Borisow N Asgari N Pitarokoili K. In Cooperation With the Neuromyelitis Optica Study G. MOG-IgG in NMO and Related Disorders: A Multicenter Study of 50 Patients. Part 1: Frequency, Syndrome Specificity, Influence of Disease Activity, Long-Term Course, Association With AQP4-IgG, and Origin. J Neuroinflamm (2016) 13(1):279. doi: 10.1186/s12974-016-0717-1 PMC5084340 27788675

[113] Inoue M Chen PH Siecinski S Li QJ Liu C Steinman L. An Interferon-Beta-Resistant and NLRP3 Inflammasome-Independent Subtype of EAE With Neuronal Damage. Nat Neurosci (2016) 19(12):1599–609. doi: 10.1038/nn.4421 PMC5482232 27820602

[114] Reder AT Feng X. How Type I Interferons Work in Multiple Sclerosis and Other Diseases: Some Unexpected Mechanisms. J Interferon Cytokine Res (2014) 34(8):589–99. doi: 10.1089/jir.2013.0158 PMC4118715 25084175

[115] Lafaille JJ Keere FV Hsu AL Baron JL Haas W Raine CS. Myelin Basic Protein-Specific T Helper 2 (Th2) Cells Cause Experimental Autoimmune Encephalomyelitis in Immunodeficient Hosts Rather Than Protect Them From the Disease. J Exp Med (1997) 186(2):307–12. doi: 10.1084/jem.186.2.307 PMC2198987 9221760

[116] Russi AE Walker-Caulfield ME Guo Y Lucchinetti CF Brown MA. Meningeal Mast Cell-T Cell Crosstalk Regulates T Cell Encephalitogenicity. J Autoimmun (2016) 73:100–10. doi: 10.1016/j.jaut.2016.06.015 PMC6364701 27396526

[117] Yin J Valin KL Dixon ML Leavenworth JW. The Role of Microglia and Macrophages in CNS Homeostasis, Autoimmunity, and Cancer. J Immunol Res (2017) 2017:5150678. doi: 10.1155/2017/5150678 PMC5749282 29410971

[118] Gordon S Taylor PR. Monocyte and Macrophage Heterogeneity. Nat Rev Immunol (2005) 5:11. doi: 10.1038/nri1733 16322748

[119] Nayak D Roth TL McGavern DB. Microglia Development and Function. Annu Rev Immunol (2014) 32:5. doi: 10.1146/annurev-immunol-032713-120240 PMC5001846 24471431

[120] Mosser DM Edwars JP. Exploring the Full Spectrum of Macrophage Activation. Nat Rev Immunol (2008) 8(12):11. doi: 10.1038/nri2448 PMC2724991 19029990

[121] Smith AM Rahman FZ Hayee B Graham SJ Marks DJB Sewell GW. Disordered Macrophage Cytokine Secretion Underlies Impaired Acute Inflammation and Bacterial Clearance in Crohn's Disease. J Exploratory Med (2009) 206(9):14. doi: 10.1084/jem.20091233 PMC2737162 19652016

[122] Rasmussen S Wang Y Kivisakk P Bronson RT Meyer M Imitola J. Persistent Activation of Microglia is Associated With Neuronal Dysfunction of Callosal Projecting Pathways and Multiple Sclerosis-Like Lesions in Relapsing–Remitting Experimental Autoimmune Encephalomyelitis. Brain (2007) 130(Pt 11):2816–29. doi: 10.1093/brain/awm219 17890734

[123] Ponomarev ED Shriver LP Maresz K Dittel BN. Microglial Cell Activation and Proliferation Precedes the Onset of CNS Autoimmunity. J Neurosci Res (2005) 81(3):374–89. doi: 10.1002/jnr.20488 15959904

[124] Lucchinetti C Bruck W Parisi J Scheithauer B Rodriguez M Lassmann H. Heterogeneity of Multiple Sclerosis Lesions: Implications for the Pathogenesis of Demyelination. Ann Neurol (2000) 47(6):707–17. doi: 10.1002/1531-8249(200006)47:6<707::aid-ana3>3.0.co;2-q 10852536

[125] MIshra MK Yong VW. Myeloid Cells — Targets of Medication in Multiple Sclerosis. Nat Rev Neurol (2016) 12:12. doi: 10.1038/nrneurol.2016.110 27514287

[126] Heppner FL Greter M Marino D Falsig J Raivich G Hovelmeyer N. Experimental Autoimmune Encephalomyelitis Repressed by Microglial Paralysis. Nat Med (2005) 11(2):146–52. doi: 10.1038/nm1177 15665833

[127] Goldmann T Wieghofer P Muller PF Wolf Y Varol D Yona S. A New Type of Microglia Gene Targeting Shows TAK1 to be Pivotal in CNS Autoimmune Inflammation. Nat Neurosci (2013) 16(11):1618–26. doi: 10.1038/nn.3531 24077561

[128] Saijo K Glass CK. Microglial Cell Origin and Phenotypes in Health and Disease. Nat Rev Immunol (2011) 11(11):775–87. doi: 10.1038/nri3086 22025055

[129] Rawji KS Yong VW. The Benefits and Detriments of Macrophages/Microglia in Models of Multiple Sclerosis. Clin Dev Immunol (2013) 2013:948976. doi: 10.1155/2013/948976 23840244 PMC3694375

[130] Wang J JS Sukhova GK Shi MA Xia M Chen H Xiang M. IgE Actions on CD4+ T Cells, Mast Cells, and Macrophages Participate in the Pathogenesis of Experimental Abdominal Aortic Aneurysms. EMBO Mol Med (2014) 6(7):17. doi: 10.15252/emmm.201303811 PMC4119357 24963147

[131] Pellizzari G Hoskin C Crescioli S Mele S Gotovina J Chiaruttini G. IgE Re-Programs Alternatively-Activated Human Macrophages Towards Pro-Inflammatory Anti-Tumoural States. EBioMedicine (2019) 43:14. doi: 10.1016/j.ebiom.2019.03.080 PMC6562024 30956175

[132] Florou D Katsara M Feehan J Dardiotis E Apostolopoulos V. Anti-CD20 Agents for Multiple Sclerosis: Spotlight on Ocrelizumab and Ofatumumab. Brain Sci (2020) 10(10):758. doi: 10.3390/brainsci10100758 PMC7589300 33092190

[133] Roll P Palanichamy A Kneitz C Dorner T Tony HP. Regeneration of B Cell Subsets After Transient B Cell Depletion Using Anti-CD20 Antibodies in Rheumatoid Arthritis. Arthritis Rheumatol (2006) 54(8):2377–86. doi: 10.1002/art.22019 16869000

[134] Coiffier B. Monoclonal Antibody as Therapy for Malignant Lymphomas. C R Biol (2006) 329(4):241–54. doi: 10.1016/j.crvi.2005.12.006 16644494

[135] van Vollenhoven RF Fleischmann RM Furst DE Lacey S Lehane PB. Longterm Safety of Rituximab: Final Report of the Rheumatoid Arthritis Global Clinical Trial Program Over 11 Years. J Rheumatol (2015) 42(10):1761–6. doi: 10.3899/jrheum.150051 26276965

[136] Ran NA Payne AS. Rituximab Therapy in Pemphigus and Other Autoantibody-Mediated Diseases. F1000Res (2017) 6:83. doi: 10.12688/f1000research.9476.1 28184292 PMC5288686

[137] Greenfield AL. Hauser Sl. B-cell Ther Multiple Sclerosis: Entering an era Ann Neurol (2018) 83(1):13–26. doi: 10.1002/ana.25119 PMC5876115 29244240

[138] Roach CA Cross AH. Anti-CD20 B Cell Treatment for Relapsing Multiple Sclerosis. Front Neurol (2020) 11:595547. doi: 10.3389/fneur.2020.595547 33551958 PMC7862116

[139] Pawluczkowycz AW Beurskens FJ Beum PV Lindorfer MA van de Winkel JG Parren PW. Binding of Submaximal C1q Promotes Complement-Dependent Cytotoxicity (CDC) of B Cells Opsonized With Anti-CD20 Mabs Ofatumumab (OFA) or Rituximab (RTX): Considerably Higher Levels of CDC are Induced by OFA Than by RTX. J Immunol (2009) 183(1):749–58. doi: 10.4049/jimmunol.0900632 19535640

[140] Pavlasova G Mraz M. The Regulation and Function of CD20: An "Enigma" of B-Cell Biology and Targeted Therapy. Haematologica (2020) 105(6):1494–506. doi: 10.3324/haematol.2019.243543 PMC7271567 32482755

[141] Fillatreau S Sweenie CH McGeachy MJ Gray D Anderton SM. B Cells Regulate Autoimmunity by Provision of IL-10. Nat Immunol (2002) 3(10):944–50. doi: 10.1038/ni833 12244307

[142] Matsushita T Yanaba K Bouaziz JD Fujimoto M Tedder TF. Regulatory B Cells Inhibit EAE Initiation in Mice While Other B Cells Promote Disease Progression. J Clin Invest (2008) 118(10):3420–30. doi: 10.1172/JCI36030 PMC2542851 18802481

